# Food Restriction Counteracts Dexamethasone-Induced Downregulation of Genes Involved in Cholesterol Homeostasis in Rat Brain during Aging

**DOI:** 10.3390/brainsci12101297

**Published:** 2022-09-26

**Authors:** Jelena Ciric, Vesna Tesic, Nikola Milovanovic, Irena Jovanovic Macura, Sanja Ivkovic, Selma Kanazir, Milka Perovic

**Affiliations:** 1 Department of Neurobiology, Institute for Biological Research “Sinisa Stankovic”—National Institute of Republic of Serbia, University of Belgrade, Bul. despota Stefana 142, 11060 Belgrade, Serbia; 2 Department of Molecular Biology and Endocrinology, “VINCA” Institute of Nuclear Sciences—National Institute of Republic of Serbia, University of Belgrade, Mike Petrovica Alasa 12-14, 11351 Vinca, Serbia

**Keywords:** every-other-day feeding, intermittent fasting, serum glucose, lipid metabolism, cortex, hippocampus

## Abstract

Glucocorticoids are the most potent anti-inflammatory agents known. Limited in vivo data are available to characterize the mechanism underlying their cognitive side effects and transient occurrence of steroid psychosis. Cholesterol is important for proper neurotransmission and brain plasticity, and disruption of its homeostasis in the brain has been closely associated with memory decline during aging and in age-related neurodegenerative disorders. In the present study, we assessed the direct effects of dexamethasone, a potent synthetic glucocorticoid, on the expression of 3-hydroxy-3-methylglutaryl coenzyme A reductase (HMGCR), apolipoprotein E (ApoE) and cholesterol 24S-hydroxylase (CYP46A1), major enzymes involved in cholesterol synthesis, metabolism, and excretion, respectively. The effects of the dexamethasone were examined during aging, in the cortex and hippocampus of 6-, 12- and 18-month-old rats, and following long-term food restriction (FR). The most prominent change observed was the age-related decrease in ApoE mRNA regardless of the food regimen applied. In animals kept on FR, this decrease was accompanied by an increase in the mRNA expression of HMGCR and CYP46A1. The present study also demonstrates that food restriction reversed most of the dexamethasone-induced changes in the expression of genes involved in regulation of cholesterol homeostasis in aging rats, in a region-specific manner.

## 1. Introduction

Glucocorticoids (GCs) are widely prescribed drugs in clinical practice due to their potent anti-inflammatory effects. Treatment with glucocorticoids, however, is usually limited by severe side effects, such as insulin resistance, dyslipidemia, osteoporosis, skin atrophy, and glaucoma, which restrict the use of higher GC dosages and their long-term application [1]. Similar metabolic effects are characteristic of disorders associated with dysregulation of the hypothalamic-pituitary-adrenal (HPA) axis and related glucocorticoid excess such as Cushing disease, obesity, diabetes, and different psychiatric disorders. Dysregulation of the HPA is also inherent to aging, additionally characterized by an exacerbated response to exogenous glucocorticoids [2]. In elderly patients, cumulative exposure to glucocorticoids aggravates cognitive impairments and hippocampal atrophy, while acute administration induces changes in mood and behavior designated as steroid psychosis [3,4].

Among the metabolic side effects of GC, mediated to a major extent by glucocorticoid receptor (GR), glucocorticoid-induced hyperglycemia and hyperlipidemia are known for decades. A significant number of studies have also shown that elevated levels of serum lipids, in particular cholesterol, may be detrimental to human learning and memory [5,6]. Microarray studies pointed towards a link between the genes involved in cholesterol homeostasis in the brain, and age-related cognitive impairments [7,8]. The ability of glucocorticoids to alter brain lipid metabolism is thus of particular importance, as this organ has the highest lipid content after adipose tissue.

Cholesterol makes up 20–30% of all lipids in the brain [9]. Although previously considered as generally structural, its pleiotropic effects on brain functioning came into focus with studies reporting its involvement in synaptic activity and neurodegenerative diseases. Namely, neuronal cholesterol depletion has been shown to induce the decrease in synaptic transmission, and further working memory deficits [10,11,12,13]. Fine alterations in cholesterol turnover in neurons appear even more important than the total brain cholesterol content itself.

Due to the presence of the blood–brain barrier, cholesterol metabolism in the brain is largely independent of that across the rest of the body [9]. Brain cholesterol is produced by de novo synthesis, and the rate-limiting enzyme, 3-hydroxy-3-methylglutaryl-coenzyme-A reductase (HMGCR) is regulated by a potent negative feedback system which affects both enzyme activity and gene expression [14]. The rate of cholesterol synthesis in the adult brain is however very low, and continuous cholesterol supply is sustained due to its extended half-life in the brain and efficient recycling by specific apolipoprotein E (ApoE) [15]. The brain also has a mechanism to prevent the detrimental excess cholesterol accumulation in the neurons [16]. Neurons express cholesterol 24-hydroxylase (CYP46A1), a member of cytochrome P450 family that converts cholesterol to oxidized 24S-hydroxycholesterol, a lipophilic molecule that passes through the BBB, enters the circulatory system and is eliminated by the liver [17].

Our previous work revealed dynamic changes in cholesterol precursors in the cortex and hippocampus during aging, and no effect on overall cholesterol content [18]. Furthermore, we found that age-related increase in the expression of HMGCR, ApoE and CYP46A1 was counteracted by the long-term food restriction (FR) [19], the intervention known to delay the aging process and improve cognitive performance [20,21]. It has been, however, clearly demonstrated that FR causes a moderate increase in circulating glucocorticoids and by this acts as a long-term mild- or low-intensity stressor which activates stress protective pathways [22]. Among different FR-induced cellular and molecular protective mechanisms, an increase in GR signaling of relevance in HPA negative-feedback regulation and further suppression of inflammation was reported in hippocampus during aging and following traumatic brain injury [23,24]. Detrimental effects of glucocorticoids can be thus ameliorated following the FR, and we therefore extended our previous research by studying the effects of dexamethasone (Dex), a potent synthetic glucocorticoid, on the expression of genes implicated in brain cholesterol homeostasis. In addition, we assessed how those changes are modulated by FR during aging in rats.

## 2. Materials and Methods

### 2.1. Animals and Treatments

A total of 50 male Wistar rats were used. All animal procedures were in compliance with the EEC Directive (86/609/EEC) on the protection of animals used for experimental and other scientific purposes, and were approved by the Ethical Committee for the Use of Laboratory Animals of the Institute for Biological Research “Siniša Stanković”, University of Belgrade. The animals were housed under standard conditions (23 ± 2 °C, relative humidity 60–70%, twelve-hour/twelve-hour light/dark cycle, *n* = 4 per cage) and their health status was routinely controlled. Food was available ad libitum(AL) until 6 months of age when the rats were divided into two groups. The AL group continued to receive food ad libitum, whereas the animals in the group on food restriction (FR) were allowed to 50% of the mean daily intake of AL animals, every other day. Both groups of rats were maintained on particular food regimen until 12 or 18 months of age, when they were divided into two additional experimental groups. Rats in control groups received a Dex vehicle solution (2% ethanol/saline; i.p.), while the animals in Dex-treated groups were injected with 4 mg/kg of dexamethasone (*n* = 5 per experimental group). Brains were isolated 18 h after Dex treatment (next morning, 9:00–10:00). The cortex and hippocampus of each animal were dissected on ice and preserved for subsequent analysis. Trunk blood was collected for serum preparation.

### 2.2. Analysis of Serum Biochemical Parameters (Glucose, Cholesterol, Triglycerides)

All measurements were taken with commercially available kits (Instrumentation Laboratory, Milan, Italy). The reagent solutions were prepared according to the manufacturer’s instructions. All assays were performed on an ILab 1800 clinical chemistry analyzer (Instrumentation Laboratory, Milan, Italy). Serum glucose was determined by a glucose-oxidase method and cholesterol and triglycerides were determined by enzymatic methods.

### 2.3. Real-Time RT-PCR

Total RNA was prepared using a Trizol reagent (Invitrogen/Thermo Fisher Scientific, Waltham, MA, USA) according to the manufacturer’s instructions. Two micrograms of RNA were further treated with 2U of RNase-free DNase I (Fermentas/Thermo Fisher Scientific, Waltham, MA, USA) and reverse transcribed using a High-Capacity cDNA Archive Kit with random hexamer primers (Applied Biosystems/Thermo Fisher Scientific, Waltham, MA, USA). The reactions were performed at 25 °C for 10 min and then at 37 °C for 2 h, in a final volume of 20 µL.

For the real-time RT-PCR analysis, the rat Assays-on-Demand Gene Expression Products (Applied Biosystems/Thermo Fisher Scientific, Waltham, MA, USA) were used. The reaction mixture (25 µL) contained 1× TaqMan Universal Master Mix with AmpErase UNG, 1× Assay Mix (Applied Biosystems/Thermo Fisher Scientific, Waltham, MA, USA) and cDNA template (1 ng of RNA converted to cDNA). PCR reactions were performed in the ABI Prism 7000 Sequence Detection System at 50 °C for 2 min, 95 °C for 10 min, followed by 40 cycles at 95 °C for 15 s and at 60 °C for 1 min. The experimental threshold was calculated as the mean baseline fluorescence signal from cycle 3 to 15 plus 10 standard deviations, and for all amplification plots the threshold cycle (Ct) was then defined. Each sample was run in triplicate and mean Ct values were used for further calculations. Validation experiments were performed as previously described [25] and consequently, glyceraldehyde 3-phosphate dehydrogenase (GAPDH) was used as a reference gene. For the effect of dexamethasone, appropriate age- and feeding regimen-matched control was used as the calibrator.

### 2.4. Statistical Analysis

All values are shown as the mean ± SEM. Statistical analysis was performed using Statistica 6.0 software (StatSoft Inc., Tulsa, OK, USA). Normality of data sets was estimated by the Shapiro–Wilk’s test, and the differences between the experimental groups were tested using nonparametric Mann–Whitney U Test. For the effect of aging, values were calculated relative to 6-month-old rat sample, and for the effects of FR feeding relative to age-matched controls. Significance was set at *p* < 0.05.

## 3. Results

### 3.1. Biochemical Parameters in Rat Serum following Dexamethasone Treatment—The Effects of Aging and FR

**Figure 1:**
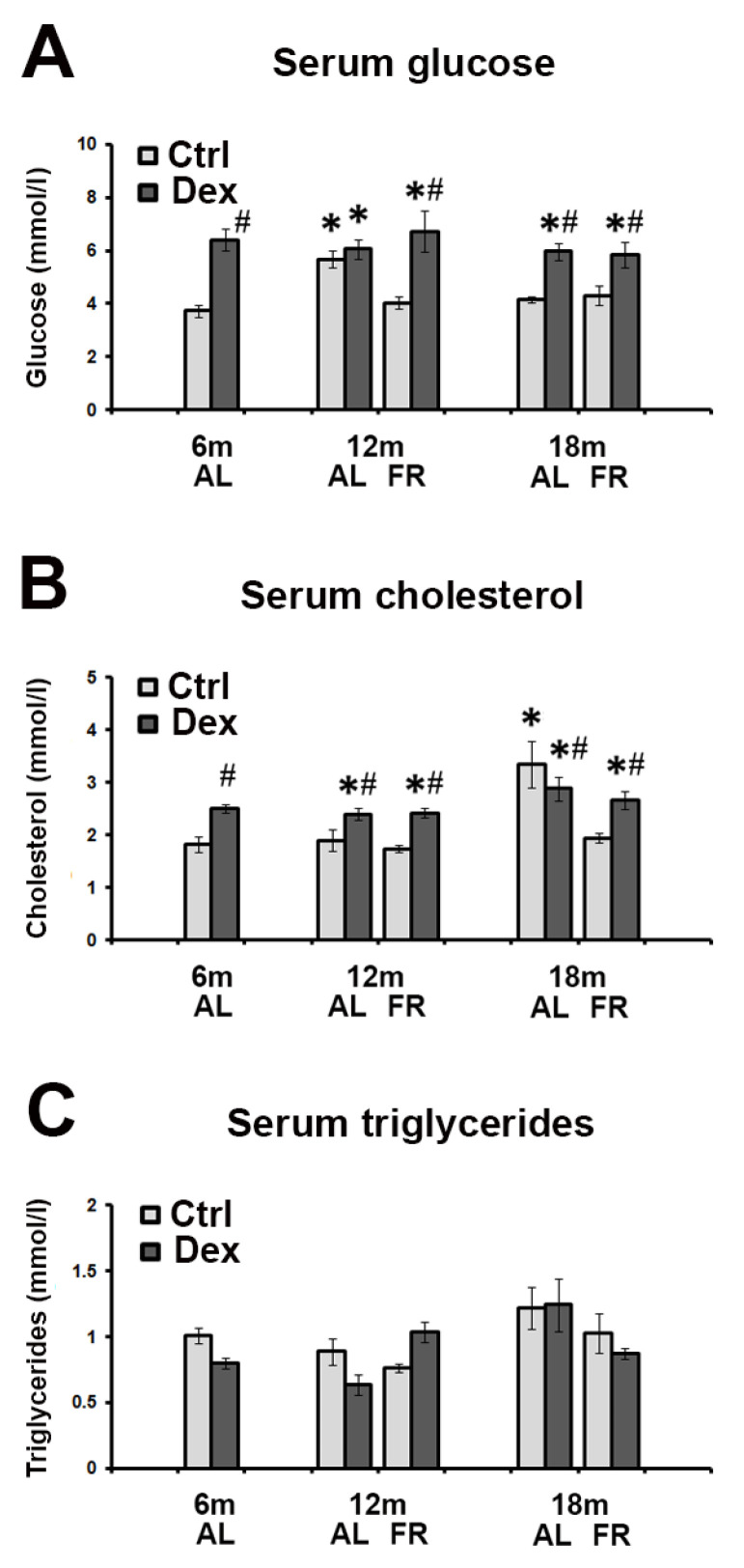
Serum levels of glucose (**A**), cholesterol (**B**) and triglycerides (**C**) in rats treated with dexamethasone (single dose, 4 mg/kg, i.p.) during aging, fed ad libitum (AL) and following food restriction (FR). Data represent the mean ± SEM (*n* = 5). * *p* < 0.05 vs. 6-month-old rats; # *p* < 0.05 vs. vehicle-treated rats.

Ad libitum-fed animals were normoglycemic and normocholesterolemic. Following FR, the level of glucose and cholesterol remained unchanged in both 12- and 18-month old animals (Figure 1A,B). Serum glucose and cholesterol levels, however, confirmed a strong systemic effect of dexamethasone. Dexamethasone caused a significant increase in the serum glucose concentration, up to 80%, in all examined groups of animals during aging in comparison to their untreated controls, except in the group of 12-month-old animals fed ad libitum. The highest increase was detected in 6-month-old animals. Serum cholesterol elevation following dexamethasone treatment was less prominent in all experimental groups, up to 40%, regardless of the age and feeding regimen applied (Figure 1B). Age-related increase observed in 18-month-old animals was reverted by FR to the values detected in 6-month-old animals. Differences between the experimental groups in the levels of triglycerides were not detected (Figure 1C).

### 3.2. FR Reversed Dexamethasone-Induced Decrease in the HMGCR mRNA Levels during Aging

**Figure 2:**
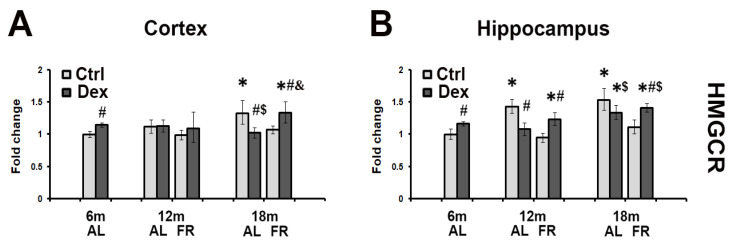
The effects of dexamethasone on HMGCR mRNA level in the cortex (**A**) and hippocampus (**B**) of rats fed ad libitum (AL; light gray bars) and following long-term food restriction (FR; dark gray bars) during aging. Dexamethasone was applied in a dose of 4 mg/kg (single i.p. injection). Values were obtained by semiquantitative qRT-PCR. Histogram represents mRNA levels expressed as ratio (fold change) calculated relative to the values obtained in 6-month-old rats. In order to obtain the differences between the experimental groups, nonparametric Mann–Whitney U Test was performed (*n* = 5 rats per group). * *p* < 0.05 vs. 6-month-old rats; # *p* < 0.05 vs. vehicle-treated rats, $ *p* < 0.05 vs. 6-month-old rats treated with Dex; & *p* < 0.05 vs. age-matched AL rats treated with Dex.

The effects of dexamethasone treatment during aging and following FR feeding regimen on the HMGCR mRNA expression profile in the rat cortex and hippocampus are presented in Figure 2. In both cortex and hippocampus of adult and 6-month-old animals, dexamethasone induced an increase in the level of HMGCR mRNA. This effect, however, was lost during aging in both regions examined. Significant reduction in HMGCR mRNA level ratio between treated and control animals, was observed in the cortex of 18-monts-old rats (30%; Figure 2A), while in the hippocampus, the most prominent decrease by 25% was detected in 12-month-old rats (Figure 2B). Food restriction counteracted those age-related effects. Regardless of the age and the brain region examined, the level of HMGCR mRNA level was increased in animals held on FR in comparison to age-matched controls. This increase led to the overall higher ratio of HMGCR mRNA than the ratio observed in 6-month-old rats, except in the cortex of 12-month-old animals (Figure 2B).

### 3.3. FR Had No Significant Effect on Age-Related Changes in ApoE mRNA Expression following the Treatment with Dexamethasone

**Figure 3:**
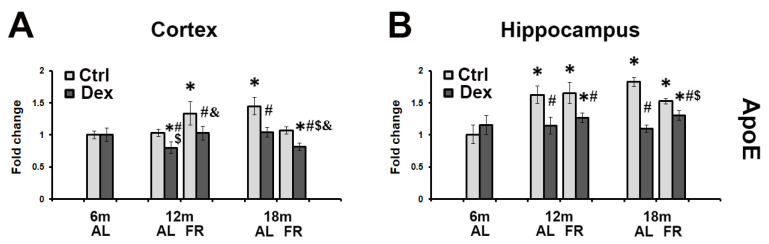
The effects of dexamethasone on ApoE mRNA level in the cortex (**A**) and hippocampus (**B**) of rats fed ad libitum (AL; light gray bars) and following long-term food restriction (FR; dark gray bars) during aging. Dexamethasone was applied in a dose of 4 mg/kg (single i.p. injection). Values were obtained by semiquantitative qRT-PCR. Histogram represents mRNA levels expressed as ratio (fold change) calculated relative to the values obtained in 6-month-old rats. In order to obtain the differences between the experimental groups, nonparametric Mann–Whitney U Test was performed (*n* = 5 rats per group). * *p* < 0.05 vs. 6-month-old rats; # *p* < 0.05 vs. vehicle-treated rats, $ *p* < 0.05 vs. 6-month-old rats treated with Dex; & *p* < 0.05 vs. age-matched AL rats treated with Dex.

In both regions examined, dexamethasone treatment had no effects on ApoE mRNA expression in the adult, 6-month-old rats (Figure 3). However, in the cortex of both 12- and 18-month-old animals, dexamethasone induced the 25–30% decrease in ApoE mRNA level regardless of the food regimen applied (Figure 3A). Age-related decrease in ApoE expression was also detected in the hippocampus where ApoE mRNA level decreased by 30 and 40% in 12- and 18-month-old AL animals, respectively (Figure 3B). A less intense ApoE mRNA decrease was observed only in the FR group of the oldest animals examined (by 20%). Nevertheless, the levels of ApoE mRNA remained below the level detected in the 6-month-old animals (Figure 3B).

### 3.4. The Effects of Dexamethasone Treatment on CYP46A1 mRNA Levels during Aging

**Figure 4:**
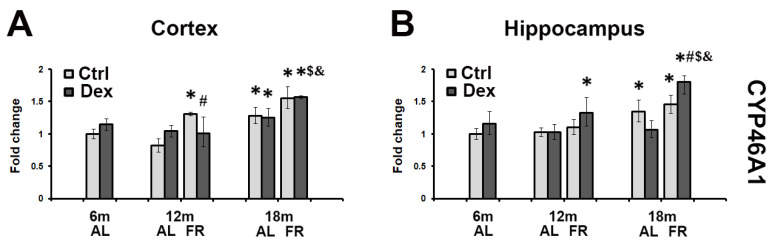
The effects of dexamethasone on CYP46A1 mRNA level in the cortex (**A**) and hippocampus (**B**) of rats fed ad libitum (AL; light gray bars) and following long-term food restriction (FR; dark gray bars) during aging. Dexamethasone was applied in a dose of 4 mg/kg (single i.p. injection). Values were obtained by semiquantitative qRT-PCR. Histogram represents mRNA levels expressed as ratio (fold change) calculated relative to the values obtained in 6-month-old rats. In order to obtain the differences between the experimental groups, nonparametric Mann–Whitney U Test was performed (*n* = 5 rats per group). * *p* < 0.05 vs. 6-month-old rats; # *p* < 0.05 vs. vehicle-treated rats, $ *p* < 0.05 vs. 6-month-old rats treated with Dex; & *p* < 0.05 vs. age-matched AL rats treated with Dex.

Dexamethasone treatment caused different age-related CYP46A1 expressional patterns in the rat cortex and hippocampus (Figure 4). The effects of FR differed as well. In adult, 6-month-old animals, no significant change in the level of CYP46A1 mRNA was observed following dexamethasone treatment. In the cortex of 12-month-old rats, however, dexamethasone induced the increase in the level of CYP46A1 mRNA by 30%, whereas in 18-month-old animals the level remained the same as the level observed in untreated controls. The only effect of FR on dexamethasone treatment was detected in early middle-aged animals where the level of mRNA decreased by 50% in comparison to untreated as well as age-matched rats (Figure 4A). In the hippocampus, age-related changes of CYP46A1 mRNA followed a similar pattern of changes to HMGCR mRNA (Figure 4B). Significant decrease by 25% observed in the group of the oldest animals was reversed following FR, by 45%, being higher in all groups of FR animals than in the age-matched control, AL group (Figure 4B).

## 4. Discussion

In the present study, we demonstrate that acute treatment with high dose of dexamethasone significantly alters lipid regulatory network in the cortex and hippocampus of rats during aging. Our results also reveal that food restriction reverses most of the dexamethasone-induced changes and that these effects are more pronounced in aged animals.

By negative feedback, dexamethasone inhibits the activity of HPA axis and the brain becomes depleted of the naturally occurring glucocorticoids, cortisol in humans and corticosterone in rats. In high doses, however, dexamethasone penetrates the blood–brain barrier [26,27] and acts as an agonist of GR [28]. Although prolonged and elevated corticosterone levels and consequent GR activation are commonly linked to neurodegeneration [29], concomitant increase in active, phosphorylated GR, its translocation to the nucleus and subsequent enhancement of its transcriptional activity following FR were associated with the increase in synaptic plasticity, reduced neurodegeneration and inflammation both during aging and following traumatic brain injury [23,24]. FR-induced elevation in serum glucocorticoids was also paradoxically found to accompany the neuroprotective effects demonstrated in several animal models of epileptic seizures, stroke, and neurodegenerative diseases [30,31,32,33]. Therefore, FR seems to activate different pathways that protect neurons from elevated levels of corticosterone itself [34].

Genes involved in cholesterol biosynthesis and uptake are tightly transcriptionally regulated in response to cellular sterol content [35], and are altered by aging [7,8,19]. Namely, cells conventionally respond to the deficits in biosynthetic sterols by increasing HMGCR level and this is particularly the case with nerve cells where cholesterol availability completely depends on the rate of its synthesis [9]. The activity of this enzyme is also considered to determine, and reflect, the overall rate of cholesterol biosynthesis in mammalian cells [36]. The rate of transcription has been further shown to be associated with its activity, implying that in 6-month-old animals dexamethasone induced an increase in cholesterol synthesis. Dexamethasone-induced increase in HMGCR transcription has been reported previously in HeLa cells, and in the liver and gut [37,38], that were further associated with its interference with the substrate availability in the sterol biosynthetic pathway [39].

Aging per se is characterized by a moderate loss of brain cholesterol (around 20%), and a decreased cholesterol synthesis rate [40]. It has been also shown that the cholesterol reduction beyond 30% is detrimental, leading to cell death [11,12]. Although concordant increase in the transcription of HMGCR during aging was reported [19,41], its short-term post-transcriptional regulation may be also involved. Namely, increased HMGCR activation was reported in aged rats and this was further directly associated with the decrease in the inhibition by phosphorylation and ROS-induced activation of p38 [42]. Dexamethasone treatment may therefore disturb the fine balance in HMGCR at multiple levels, contributing to altered membrane composition and related functional impairments reported in the aging brain [43,44]. Data obtained for rats treated with dexamethasone indeed revealed a continuous age-related decrease in the levels of cortical and hippocampal HMG-CoA reductase mRNA.

The inability of the aged system to maintain lipid homeostasis is further supported by a substantial decrease in ApoE mRNA that were not reversed even by FR. ApoE is essential for brain lipid transport and thus has a role in neuronal membrane maintenance, repair, and consequently in learning and memory processes [45]. Numerous studies have shown a region-specific transcriptional upregulation of ApoE in aged animals [8,19,46] implying a partial compensation for age-related degenerative processes that can be also further disturbed with the dexamethasone. In line with this, a negative regulatory element showing homology with a sterol responsive element was identified in the ApoE promoter [47].

A dexamethasone–induced ApoE downregulation may however imply local cholesterol accumulation, which was also shown to be detrimental [16]. We therefore further analyzed the effects of acute dexamethasone treatment on gene expression of CYP46A1 involved in the most important mechanism by which the brain eliminates cholesterol excess. In contrast to strictly synchronized synthesis and reuptake, cholesterol elimination appears to be much less regulated. Expression of CYP46A1 has been shown to be affected by age and oxidative stress, but the availability of cholesterol substrate does not seem to be critical in the transcriptional regulation of this enzyme [17,48]. Resistance to the regulation was shown for various hormones and oxysterols, and CYP46A1 promoter activity in vitro was induced only by combined treatment with dexamethasone and interleukin-6 [48]. Studies performed in the CYP46A1^−/−^ mouse further revealed that the steady-state level of brain cholesterol is actually maintained by the reduction in cholesterol synthesis [17]. This suggests that the strict balance between the synthesis and elimination is the most important factor for the cholesterol homeostasis in the adult brain. We also observed a direct correlation between the transcriptional regulation of HMGCR and CYP46A1 in the hippocampus regardless of the age or food regimen applied. In the cortex, however, feedback mechanism efficiency was different in aged animals—the CYP46A1 levels were not changed although the levels of HMGCR were decreased. Increases in CYP46A1 expression are generally viewed as beneficial in different types of CNS injury as increased cholesterol clearance is involved in elimination of cellular debris which in turn causes both a reduction in inflammation and a reduction in the production of free radicals [49,50].

It is also known that long-term FR also induces a significant reduction in cholesterol synthesis and the decrease in the level of HMGCR in the cortex and hippocampus and even in the cerebellum of rats [19,36,51]. Proper regulation of cholesterol turnover may thus represent one of the mechanisms by which FR achieves its protective effects in the aged brain. Substantial impairment of ApoE mediated transport following dexamethasone treatment may be compensated by a parallel increase in the amount of HMGCR and CYP46A1. However, increase in cholesterol turnover across the brain has been also shown in neurodegenerative disorders such as Alzheimer’s disease (AD) and Niemann-Pick type C disease although for AD it has been shown that the rate of turnover lowers as the disease progresses [52]. On the other hand, enhancement of the cholesterol turnover in the brain has been reported to improve cognition in AD mouse models [53].

Interestingly, changes observed for HMGCR and CYP46A1 in the hippocampus had the same pattern as those observed for total serum cholesterol. Although the direct association is still not clear, a significant number of studies show that elevated serum cholesterol is a risk factor for mild cognitive impairment and dementia [5,6]. Excess serum cholesterol, including the dexamethasone-induced one, can induce peripheral pathology which can affect the brain via cholesterol metabolites, pro-inflammatory mediators and antioxidants. Together with the glucocorticoid-induced hypercholesterolemia, this can facilitate transport and delivery of lipophilic compounds, such as dexamethasone, and in turn, can cause additional profound local metabolic changes aggravating cognitive deficits, including the development of steroid psychosis.

## 5. Conclusions

The results of the present study further elucidate the link between hypercholesterolemia, brain expression of genes involved in cholesterol homeostasis and aging. Dexamethasone-induced changes are of relevance for numerous side effects affecting proper brain functioning and cognitive impairments associated with glucocorticoid therapy. Although the lack of relevant protein levels may be considered as study limitation and warrant further studies, comprehensive mRNA analysis is crucial for systematic characterization of the mechanism underlying the cognitive side effects of glucocorticoids and transient occurrence of steroid psychosis. We here also present the evidence that FR ameliorates the dexamethasone-induced effects providing thus new insight into the beneficial effect of FR in the brain.

## Data Availability

Not applicable.
